# Is Acceleration Used for Ocular Pursuit and Spatial Estimation during Prediction Motion?

**DOI:** 10.1371/journal.pone.0063382

**Published:** 2013-05-16

**Authors:** Simon J. Bennett, Nicolas Benguigui

**Affiliations:** 1 Research Institute for Sport & Exercise Sciences, Liverpool John Moores University, Liverpool, United Kingdom; 2 Normandie Université, Caen, France; 3 UNICAEN, CESAMS (EA 4260), Caen, France; VU University Amsterdam, The Netherlands

## Abstract

Here we examined ocular pursuit and spatial estimation in a linear prediction motion task that emphasized extrapolation of occluded accelerative object motion. Results from the ocular response up to occlusion showed that there was evidence in the eye position, velocity and acceleration data that participants were attempting to pursue the moving object in accord with the veridical motion properties. They then attempted to maintain ocular pursuit of the randomly-ordered accelerative object motion during occlusion but this was not ideal, and resulted in undershoot of eye position and velocity at the moment of object reappearance. In spatial estimation there was a general bias, with participants less likely to report object reappearance being behind than ahead of the expected position. In addition, participants’ spatial estimation did not take into account the effects of object acceleration. Logistic regression indicated that spatial estimation was best predicted for the majority of participants by the difference between actual object reappearance position and an extrapolation based on pre-occlusion velocity. In combination, and in light of previous work, we interpret these findings as showing that eye movements are scaled in accord with the effects of object acceleration but do not directly specify information for accurate spatial estimation in prediction motion.

## Introduction

In our everyday surrounds objects are often transiently occluded, for example when a cyclist rides past stationary vehicles, or during a free-kick in soccer the ball moves behind teammates and/or opponents. In such instances, it is necessary for the road user or player to extrapolate the unseen trajectory in order to then act appropriately upon object reappearance (e.g., avoid a collision or make an interception). In the laboratory, researchers have attempted to determine the extent to which eye movements contribute to the extrapolation process, and thereby the estimation of timing [1]–[4]. In general, findings indicate that participants make more accurate temporal estimation when they are permitted to move their eyes compared to when they are fixating. Analysis of eye movements when permitted to pursue an object that does not reappear after occlusion has indicated that participants do so with a combination of smooth pursuit and saccades [5]. However, rather than matching eye to object motion throughout occlusion, participants make a large amplitude saccade to the arrival location and then wait a variable interval before making their temporal estimation; for similar findings see [2]. Accordingly, temporal estimation was found to be based on visual properties of the stimulus prior to object occlusion, and not when the eyes arrived at the point of contact. Rather than extra-retinal input available from eye movements having predictive value, a reasonable explanation is that ocular pursuit of the object prior to occlusion facilitates velocity perception [6], which influences temporal estimation accuracy.

Two important features of the stimulus used in the temporal prediction motion task, are that: i) the object does not reappear when it reaches the arrival location, and ii) a visual cue representing the arrival location remains present throughout the presentation. Together, these stimulus features reduce the ability and need to match eye position and velocity to that of the object during occlusion, and thereby the potential contribution of oculomotor information to estimation accuracy. For instance, because the object remains occluded throughout, there is no need to minimize position error and retinal slip (i.e., measures of response effectiveness) that would otherwise be available later in the trajectory. In addition, the presence of a visual cue at the arrival location acts as an attractor to which participants move the eyes shortly after occlusion of the moving object [2], [5]. The contribution from eye movements during occlusion in temporal prediction motion could also be influenced by the use of cognitive strategies [4], [7]. For instance, participants might perceive information related to the properties of the moving object (e.g., velocity and occlusion distance), and then count down the time from object occlusion to arrival at the point of contact [8], which would not be dependent on continued pursuit. Such a strategy would also be negatively affected by a misperception of stimulus properties during the initial visible part of the trajectory, and hence could explain the temporal estimation error observed during fixation [1], [3], as well as with accelerating objects [5], [9], and the presence of distractors [7], [10], [11].

The processes involved in extrapolating an occluded trajectory have also been examined by requiring participants to make a spatial estimation in the absence of a fixed visual cue at the reappearance position (i.e., interruption paradigm). Response accuracy for spatial prediction motion (i.e., the task of the interruption paradigm) places greater demand on extrapolation of the occluded trajectory because the participant does not know in advance where and when the object will reappear; for different models of the extrapolation process see [10]. In such a task, participants combine smooth pursuit and saccades during occlusion to extrapolate well with their eyes the moving object trajectory until its reappearance [12]. More recently, it has been reported that accuracy of estimating whether an occluded object reappeared early or late during occlusion (i.e., based on comparison of actual to expected position) was better when participants were encouraged to maintain pursuit compared to when they were fixating [2]. In addition, accuracy of estimating early, but not late, object reappearance, improved as a function of eye position accuracy during the initial 360 ms of occlusion. The implication, therefore, is that ocular pursuit can impact upon spatial estimation accuracy, not only because it influences velocity perception and locates the eyes in the vicinity of object reappearance, but also because extra-retinal input from eye movements could be used as a reference for occluded object motion [13].

In the current experiment, we further examined eye movements and spatial estimation in a prediction motion task where the object could undergo negative or positive acceleration. The use of accelerating objects in the spatial prediction motion task, where there is an absence of contextual cues regarding reappearance location, was important because it encouraged extrapolation of the occluded trajectory. Also, such motion is more representative of that experienced in everyday life where objects are influenced by gravitational and frictional forces, and therefore do not typically have constant velocity. It was expected that participants would initially scale pursuit to the object velocity generated by the different levels of object acceleration [14]–[16], and then attempt to maintain the ocular response during occlusion using a combination of reduced-gain smooth pursuit and saccades. Given oculomotor sensitivity to the object motion properties prior to occlusion, it was anticipated that this would also be reflected in the eye movements at the moment of object reappearance [17], [18]. In addition, if the veridical properties of object motion prior to occlusion were also taken into account for spatial estimation, no systematic error should be expected. On the other hand, not taking into account the effects of object acceleration, which has been observed in temporal prediction motion [5], [9], should result in overestimation of the occluded distance for a negatively accelerating object and underestimation for a positively accelerating object.

Having examined separately the perceptual and oculomotor response, logistic regression was then used to determine if individual participant’s spatial estimation could be predicted by a position error signal related to the eyes or a mental extrapolation (i.e., internal cognitive model) of the occluded object motion. Extending upon recent work [2], we examined the predictive value of variables available at the moment of object reappearance because this is when spatial estimation occurs, and as such is likely to provide more salient information than pursuit accuracy prior to and around occlusion. In combination, the above analyses sought to determine the contribution of eye movements during occlusion to spatial estimation in prediction motion, and therefore add to understanding of the processes involved in motion extrapolation.

## Methods

### Participants

Ten human male participants (mean age: 24 years) completed the experiment. Participants had varying levels of experience of oculomotor experiments but all were familiarized to the current task and procedure. Participants were instructed that they be required to pursue objects with different motion characteristics, which would undergo transient occlusion (see below for more detail). Except for one participant, who was an author (SJB), none were aware of the different levels of acceleration or the number of position steps. All participants had normal or corrected-to-normal vision, were healthy and without any known oculomotor abnormalities. Written consent was obtained before the experiment, and in accordance with the Declaration of Helsinki, the protocol was approved by the Liverpool John Moores University local ethics committee.

### Apparatus

Participants sat in a purpose-built dark room, facing a flat white screen (2.0×1.7 m) at a viewing distance of 1.9 m. The head was supported with a height-adjustable chin rest and a pad placed at the nape of the participant’s neck. Experimental stimuli were generated on a host PC (Dell Precision 670) using the COGENT toolbox implemented in MATLAB (Mathworks Inc) and displayed on the screen using a CRT projector (Barco Graphics 908). The stimuli were presented with a spatial resolution of 1024×768 pixels and a refresh rate of 85 Hz. Estimation of reappearance position was determined from the button pressed (left = behind, right = ahead) on a laser mouse (Logitech G5). Movement of both eyes was recorded at 200 Hz using a Chronos 3D eye tracker (Chronos Vision). Only data from the left eye were stored to a target PC for off-line analysis using proprietary routines developed in Matlab.

### Task and Procedure

**Figure 1:**
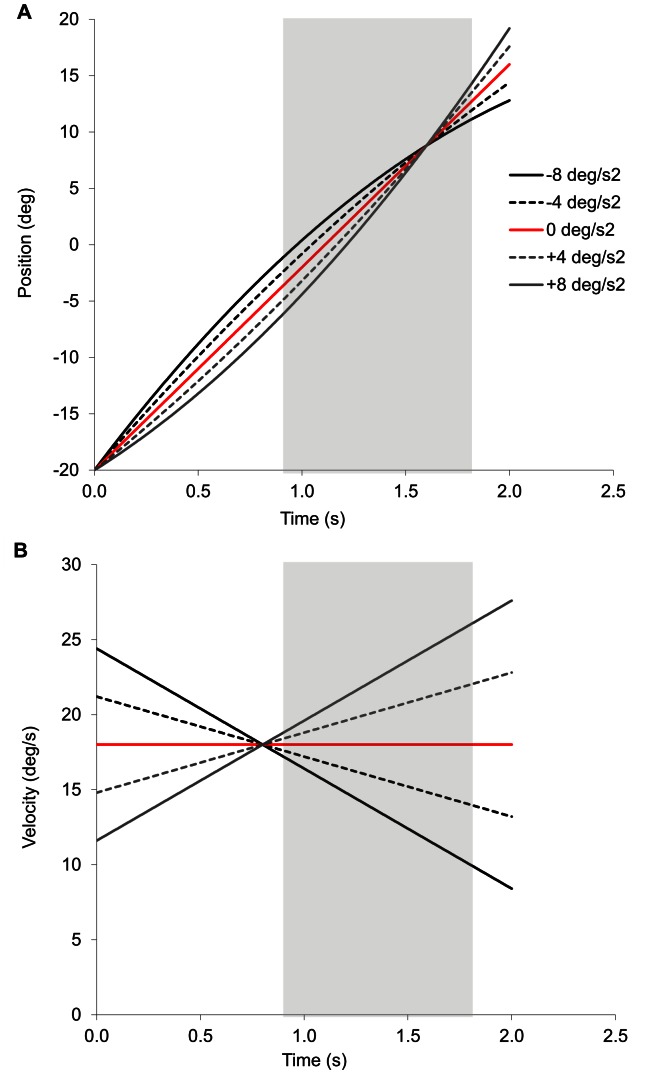
Object motion characteristics. Position (panel A) and velocity (panel B) are shown for the different levels of acceleration (see legend) as a function of time normalized to motion onset. Light grey shaded bars represent occlusion.

Participants were required to make a spatial estimation regarding the horizontal reappearance position of an occluded moving object (see Figure 1A and 1B). Each trial began with the appearance of a green spherical object (0.6 deg diameter), which was always located at −20 deg to the left of, the participant’s point of observation. After a fixed duration of 1500 ms the green spherical object changed color to red, which signaled to the participant that it would soon begin to move. Then following a random foreperiod between 1650 and 1850 ms, the red spherical object moved horizontally for 800 ms from the left to the right. Initial velocity was either 24.4, 21.2, 18.0, 14.8, or 11.6 deg/s, and was uniquely matched with a single level of acceleration (−8, −4, 0, +4, or +8 deg/s^2^, respectively) such that pre-occlusion object velocity was 18.0 deg/s. With these parameters, pre-occlusion velocity did not uniquely specify reappearance position and velocity, and thus had limited predictive value. Similarly, while initial velocity was negatively correlated with reappearance velocity, it was not correlated with reappearance position, and also had an instantaneous value that would unlikely be perceived in a single frame of presentation (i.e., 11.76 ms). On the other hand, change in velocity resulting from the outermost levels of acceleration (−8 and +8 deg/s^2^) during the initial 800 ms of motion was above the accepted 25% discrimination threshold [19], [20]. Therefore, although not perceived directly from acceleration sensitive cells, the fact that the initial visible part of the trajectory was longer 100–140 ms [21] meant that a veridical acceleration signal could have been reconstructed from population coding of velocity sensitive cells in area MT [16], [22]–[24]. After occlusion the object continued to move, unseen, horizontally across the screen for 800 ms. It then reappeared with a position step that was either behind or ahead (−5, −3, −1, +1, +3, +5 deg) of the veridical position had the object continued to move with the same motion properties. Using these parameters, object displacement differed as a function of object acceleration but veridical reappearance position was constant at 8.8 deg to the right of screen centre. In this way, the inclusion of a position step resulted in only 5 actual reappearance positions (3.8, 5.8, 8.8, 11.8, 13.8 deg), thus also minimizing this as a cue to infer occluded object motion properties.

Each participant performed a total of 165 trials that were received in a single experimental session lasting approximately one hour. The first block of 15 trials was used as a familiarization session and was not included in the analysis. Each subsequent block comprised 30 experimental trials, 1 for each combination of reappearance step (−5, −3, −1, +1, +3, +5 deg) and object acceleration (−8, −4, 0, +4, +8 deg/s^2^), received in a pseudo-random order. Participants were instructed to track the moving object with their eyes for the entirety of the presentation and estimate its reappearance relative to the expected position had it continued to move with the same motion properties throughout. Object reappearance was always subject to a position step, hence requiring participants to make a two-alternative, forced-choice estimation. No feedback was given regarding estimation error in order to emphasize use of veridical motion properties and thereby minimize the likelihood of participants responding based on a learned heuristic.

### Data Analysis

For each trial, the mouse button data was used to determine whether participants estimated the actual reappearance position to be behind (left mouse click) or ahead (right mouse click) of the expected reappearance position. The proportion of trials estimated as “behind” was then calculated for each combination of object parameters [10] and subjected to arcsine transformation to ensure a normal distribution. For the eye movement data, eye position was low-pass filtered at 25 Hz and then differentiated by means of a central difference algorithm to derive eye velocity and acceleration. Eye acceleration data was then scanned to determine the presence of saccades. Saccade onset was detected when eye acceleration was beyond a threshold of 750°/s^2^. When the threshold criteria were exceeded the complete saccade trajectory was identified by finding the peak and trough of acceleration; saccade offset was detected when eye acceleration after the trough was greater than −750°/s^2^. Identified saccades, plus an additional five data points (equivalent to 25 ms) at the beginning and end of the saccade trajectory, were removed and replaced by a linear interpolation routine based on the smooth eye velocity before and after the saccade [25]. From these data, we extracted from each trial the eye position and velocity at the start and end of occlusion, as well as a measure of eye acceleration. The latter was derived by calculating the slope of velocity data from 5 samples either side of the start and end of occlusion.

For eye movement and spatial estimation data, intra-participant means from the 5 experimental trials per combination of stimulus parameters were calculated and submitted to separate 6 reappearance step (−5, −3, −1, +1, +3, +5 deg)×5 acceleration (−8, −4, 0, +4, +8 deg/s^2^) ANOVA with repeated measures on both factors. Within-subject contrasts were used to determine if there was a significant linear trend for each factor. If present, Holm-Bonferroni corrected comparisons were restricted to pairs of equal and opposite sign of the independent variables (e.g., −5 deg step vs. +5 deg step, or −8 deg/s^2^ vs. +8 deg/s^2^) in order to control for familywise error rate while maintaining acceptable statistical power.

To examine the information used as the basis of spatial estimation, individual participant data were submitted to separate logistic regression analysis. The proportion of behind judgments was the dependent variable, while as predictors we included variables available at the moment the object reappeared calculated with respect to the object or the eye. For predictors related to the object trajectory, we used the difference between an extrapolation of object position based on veridical motion properties (i.e., −5, −3, −1, +1, +3, +5 deg) or pre-occlusion velocity (see Table 1). For a predictor based on eye movement, we calculated for each level of object acceleration and reappearance step, the mean difference (i.e., across the 5 trials) between eye position and object position at reappearance.

**Table 1:** Difference between object reappearance position and an extrapolation that took into account the effects of acceleration (veridical), or pre-occlusion velocity (PreVel).

Veridical	PreVel −8	PreVel −4	PreVel 0	PreVel +4	PreVel +8
−5	−7.56	−6.28	−5	−3.72	−2.44
−3	−5.56	−4.28	−3	−1.72	−0.44
−1	−3.56	−2.28	−1	0.28	1.56
1	−1.56	−0.28	1	2.28	3.56
3	0.44	1.72	3	4.28	5.56
5	2.44	3.72	5	6.28	7.56

## Results

### Eye Movements

**Figure 2:**
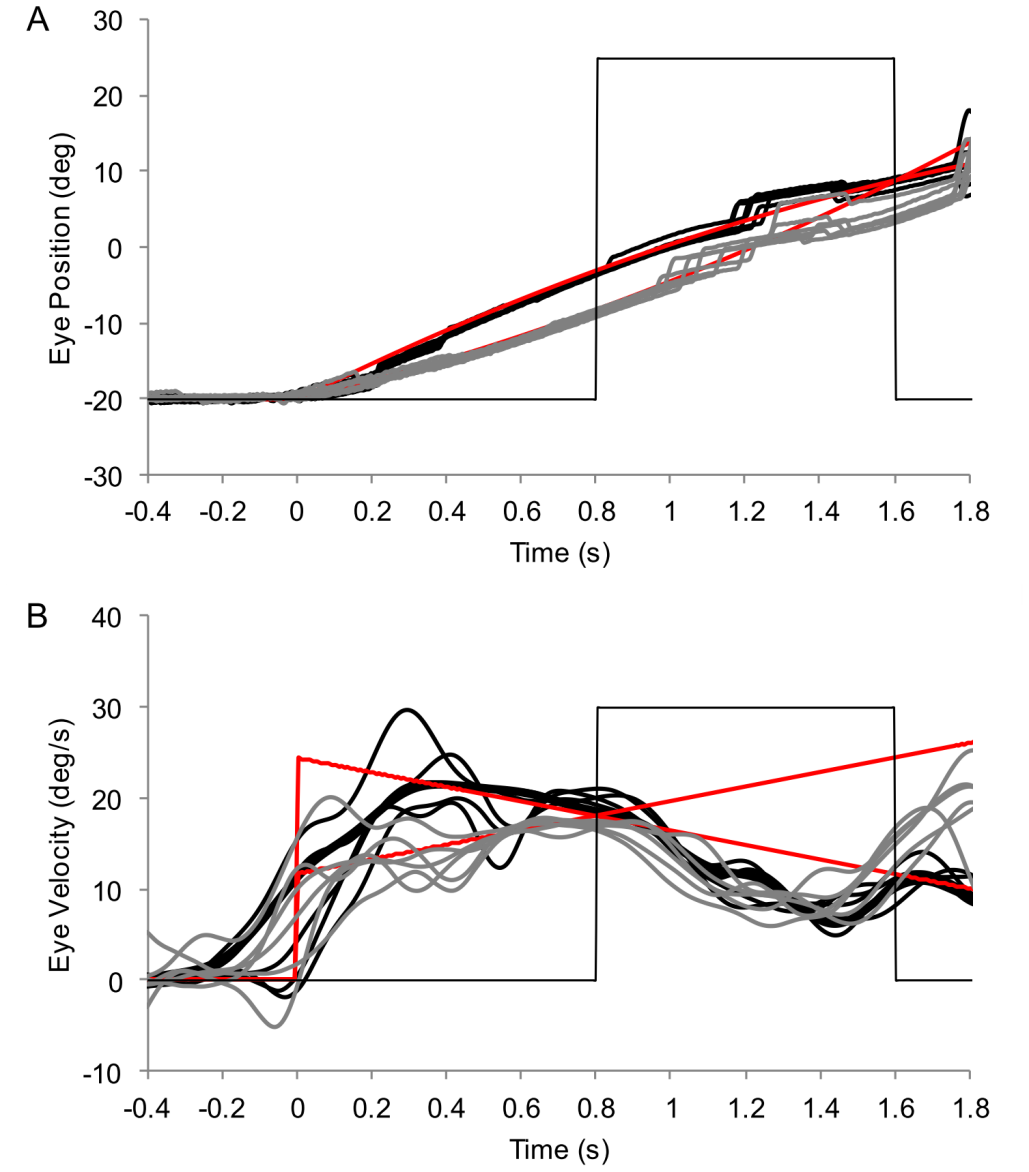
Representative eye position (panel A) and velocity (panel B). Data are shown from a single participant in trials where the object accelerated at −8 (black lines) and +8 (grey lines) deg/s^2^. Red lines represent object position and velocity, respectively. Thin grey line depicts when the object is visible (low) and occluded (high).

**Figure 3:**
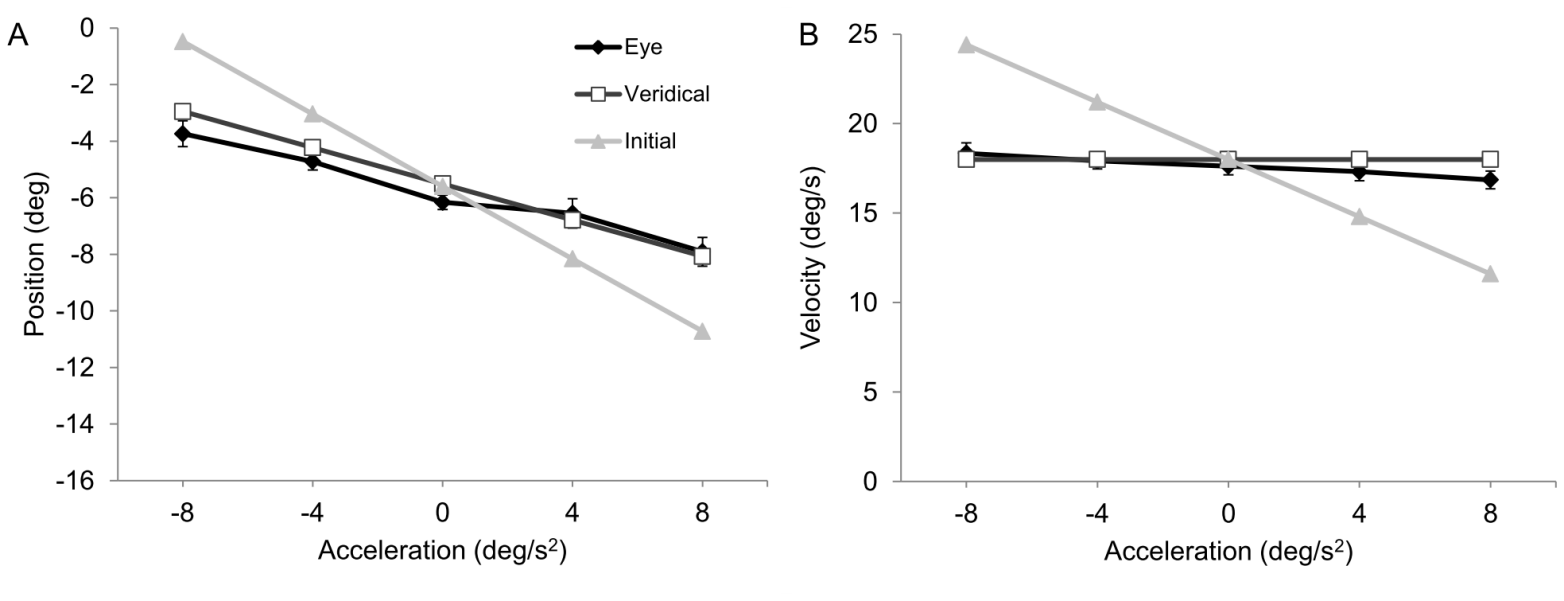
Group mean eye position (panel A) and velocity (panel B) at disappearance (filled black diamonds on black line). Object position and velocity data at disappearance of actual object motion is represented by filled white squares on black line. Also shown for comparison are position and velocity based on extrapolation of initial object velocity (filled grey triangles on grey line). Capped bars show standard error of the mean.

Figure 2 shows typical eye movement data in trials where the object accelerated at −8 and +8 deg/s^2^. Participants initially pursued the moving object with a combination of smooth and saccadic eye movements that brought the eye close to the object’s position, velocity and acceleration at the moment of occlusion. This resulted in a main effect of acceleration on eye position [F(4,36) = 48.92, p<.01, η_p_^2^ = 0.84], velocity [F(4,36) = 11.3, p<.01, η_p_^2^ = 0.56] and acceleration [F(4,36) = 12.6, p<.01, η_p_^2^ = 0.58]. Within-subject contrasts indicated a significant linear trend for eye position [F(1,9) = 59.1, p<.01] and acceleration [F(1,9) = 47.9, p<.01], which both changed in the expected direction as a function of object acceleration. In addition, eye position and acceleration differed in the pairwise comparison between object accelerations of −8 and +8 deg/s^2^, as well as −4 and +4 deg/s^2^ (see Figure 3A). Eye velocity deviated from pre-occlusion object velocity (18 deg/s), becoming lower as object acceleration changed from negative to positive [F(4,36) = 11.3, p<.01, η_p_^2^ = 0.56]. This resulted in a significant linear trend [F(1,9) = 25.82, p<.01], as well as a difference in the pairwise comparison between object accelerations of −8 and 8 deg/s^2^ [p<.01] (see Figure 3B). Importantly, though, across each combination of object acceleration and step, the group mean difference between eye and object velocity was no greater than 1.4 deg/s, which is much less than would be expected had participants attempted to track the initial object velocity. Together, these data confirm that participants were attempting to track the object during the initial part of the trajectory in accord with position and velocity generated by the different levels of acceleration [15], [17].

**Figure 4:**
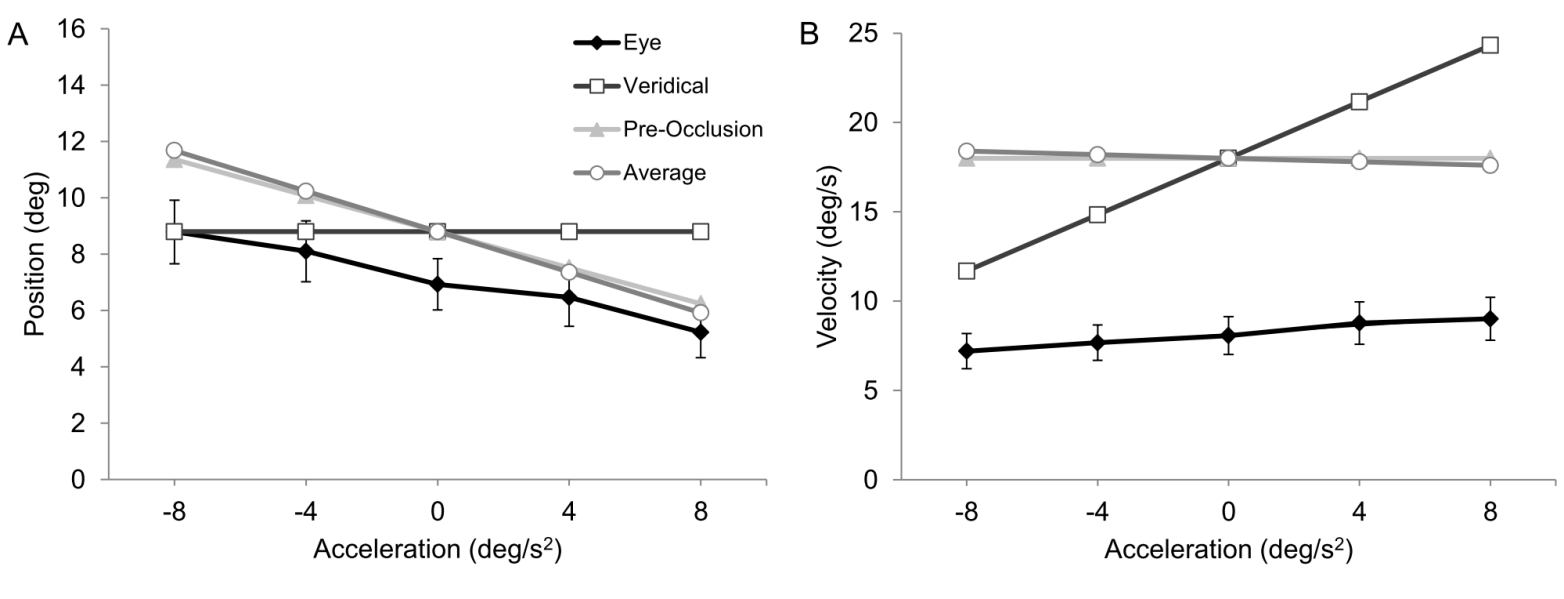
Group mean eye position (panel A) and velocity (panel B) at reappearance (filled black diamonds on black line). Object position and velocity data at reappearance of actual object motion is represented by filled white squares on black line. Also shown for comparison are extrapolated position and velocity based on pre-occlusion velocity (filled grey triangles on grey line) and average velocity of the final 100 ms of object motion extrapolation (filled white circles on grey line). Capped bars show standard error of the mean.

After the object disappeared, participants continued to move their eyes using a combination of smooth pursuit and saccades. Eye velocity initially decayed in the absence of visual feedback and hence deviated away from object velocity in trials with 0, +4 or +8 deg/s^2^ acceleration. Subsequently, participants often exhibited an anticipatory increase in eye velocity toward the end of the occlusion [18]. This was not the case when for objects with negative acceleration, resulting in a decaying eye velocity that matched well the reducing object velocity. ANOVA indicated a main effect of acceleration on eye position [F(4,36) = 18.5, p<.01, η_p_^2^ = 0.67], and eye velocity [F(4,36) = 14.3, p<.01, η_p_^2^ = 0.61] at object reappearance. There was a negative linear relationship between eye position and object acceleration [F(1,9) = 22.1, p<.01]. Pairwise comparison indicated differences in eye position between object accelerations of −8 and +8 deg/s^2^ [p<.01] and −4 and +4 deg/s^2^ [p<.01] (see Figure 4A). Eye velocity, however, increased in line with object velocity for the different levels of object acceleration. Specifically, there was a significant linear scaling of eye to object velocity at reappearance [F(1,9) = 25.0, p<.01], as well as a difference in the pairwise comparison between object accelerations of −8 and +8 deg/s^2^ [p<.01] and −4 and +4 deg/s^2^ [p<.01] (see Figure 4B). Importantly, the linear trend in eye velocity at reappearance was not a simple extension of pre-occlusion eye velocity. Nor was it consistent with extrapolation based on pre-occlusion velocity or an average estimate (i.e., negative slope). Therefore, although not ideal, the scaling in eye velocity at reappearance would appear to be reflective of the change in object velocity caused by object acceleration throughout the initial visible part of the trajectory. This was confirmed by regression analysis on the individual-participant eye velocity data against object reappearance velocity. As shown in Table 2, the slope of the regression line was significantly different from zero for 7 of the 10 participants. It was negative for 1 participant but the relationship was not significant. Further evidence that participants were attempting to extrapolate in accord with veridical object motion properties was indicated by a main effect of acceleration in a subsidiary analysis of change in eye velocity between pre-occlusion and reappearance [F(4,36) = 21.47, p<.01, η_p_^2^ = 0.71], and eye displacement during occlusion [F(4,36) = 2.8, p<.05, η_p_^2^ = 0.23]. For both measures, there was a linear increase as object acceleration change from negative to positive [p<.05]. Also, for change in eye velocity between pre-occlusion and reappearance, pairwise comparison indicated differences between object accelerations of −8 and +8 deg/s^2^ [p<.01] and −4 and +4 deg/s^2^ [p<.01]. Participants exhibited a smaller change in eye velocity between pre-occlusion and reappearance for positive compared to negative object accelerations.

**Table 2:** Results (slope, intercept,R^2^ and p value) of individual-participant (P) linear regression between eye velocity at reappearance and reappearance object velocity predicted from veridical motion properties.

P	Slope	Intercept	R^2^	p
1	0.16	1.25	0.37	0.001
2	−0.01	5.25	0.01	0.685
3	0.19	10.28	0.45	0.001
4	0.30	7.05	0.61	0.001
5	0.25	5.56	0.53	0.001
6	0.16	6.74	0.32	0.001
7	0.20	3.52	0.65	0.001
8	0.12	3.28	0.14	0.041
9	0.08	6.60	0.09	0.101
10	0.04	5.06	0.02	0.480

### Spatial Estimation

**Figure 5:**
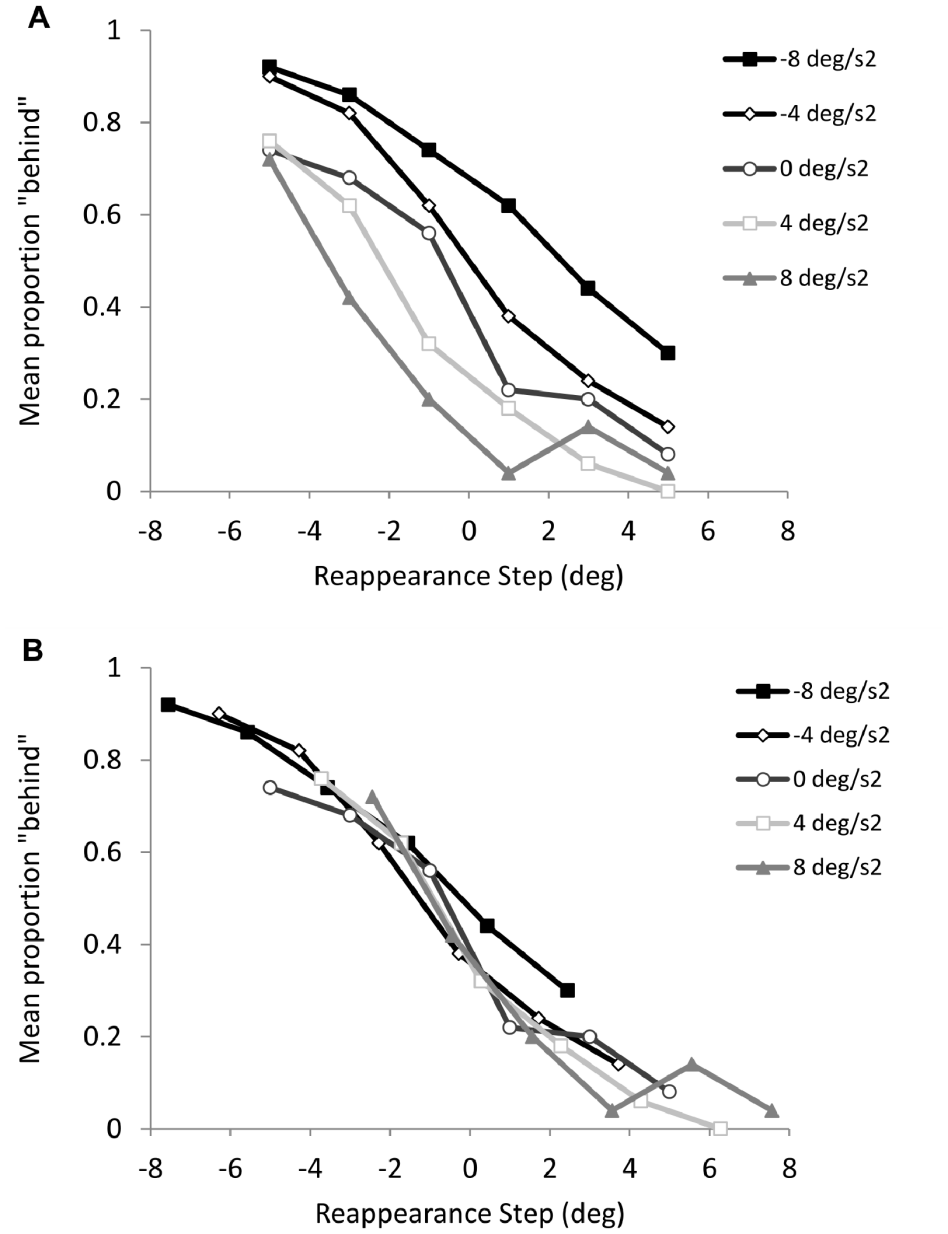
Group mean proportion of trials with reappearance position judged behind expected position based on veridical (panel A) or pre-occlusion velocity (panel B) extrapolation.

There was a main effect of reappearance step [F(5,45) = 32.70, p<.01, η_p_^2^ = 0.78] and acceleration [F(4,36) = 21.66, p<.01, η_p_^2^ = 0.71]. Within-subject contrasts indicated a significant linear trend for both independent variables; F(1,9) = 44.35, p<.01 and F(1,9) = 31.18, p<.01. Collapsed across the different levels of object acceleration, participants were most errorful for objects reappearing at −1 deg, resulting in a correct response that did not differ from chance level [p>0.01]. There was also a significant interaction between step and acceleration [F(20,180) = 1.98, p<.01, η_p_^2^ = 0.18]. As can be seen in Figure 5A, with constant velocity objects (i.e., baseline – grey line with solid circles) there was a tendency for participants’ to exhibit more errorful spatial estimation (i.e., closer to 0.5 probability) for objects reappearing with negative position step. This was confirmed by single-sample T-tests, which showed estimation of constant velocity objects differed from chance only when they reappeared with positive step [p<0.01]. Compared to this baseline, it can also be seen that objects with positive acceleration were estimated to reappear behind less often (i.e., underestimating extrapolated position), whereas the opposite was evident for objects with negative acceleration (i.e., overestimating extrapolated position). Not surprisingly, logistic curve fitting on the group mean data returned PSE that differed in accord with object acceleration (see Table 3). In combination, these data indicate a general tendency to underestimate the occluded distance of constant velocity objects, and a lack of sensitivity to the effects of acceleration. Next, we plotted the spatial estimation data against the reappearance error that would have been evident had participants extrapolated the occluded trajectory based on pre-occlusion velocity. As can be seen Figure 5B, the spatial estimation data overlapped for each level of object acceleration. This was also evident from logistic curve fitting on the group mean data, which returned PSE that were very similar for each level of object acceleration (see Table 3).

**Table 3:** Point of subjective equality (PSE) for spatial estimation based on veridical (upper panel) or pre-occlusion velocity (lower panel) extrapolation.

Acceleration	Veridical	PreVel
−8	2.37	−0.19
−4	0.23	−1.05
0	−1.31	−1.31
4	−2.36	−1.08
8	−3.54	−0.98

### Information for Spatial Estimation

**Figure 6:**
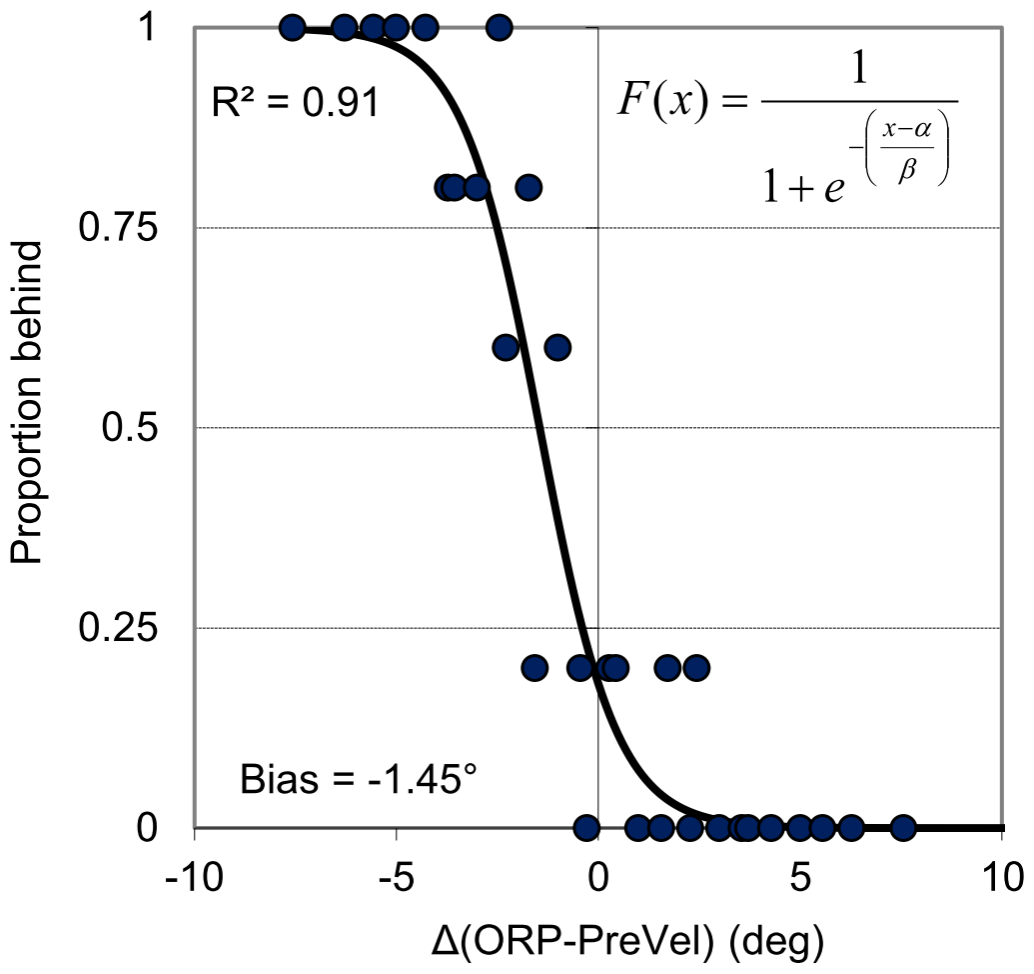
Individual-participant (P2) logistic regression function fitted to spatial estimation data plotted against the difference between object reappearance position (ORP) and an extrapolation of the occluded object position based on pre-occlusion velocity (PreVel). JND was 0.96 and bias (PSE) was −1.45.

As can be seen from the results on logistic regression conducted on individual participant data (Table 4), the difference between object reappearance position and an extrapolation of object position based on pre-occlusion velocity was the best predictor of spatial estimation for eight participants. The best predictor for the other two participants was the difference between object reappearance position and eye position. Dependent t-test on z-transformed correlation coefficients from the logistic regression indicated that the difference between object reappearance position and an extrapolation of object position based on pre-occlusion velocity was the best predictor of spatial estimation for the group of participants, t(9) = 2.57, p<.05. In terms of individual participant bias (i.e., PSE), which is a measure of how far away from the ideal value of zero is from 0.5 response accuracy (see Figure 6), there was an equal split of negative and positive values across participants, which were seemingly unrelated to the information returned as the best predictor. For individual participant JND, which here reflects the minimum detectable difference between object and extrapolated position, there was a range of −7.07 to −0.96 across participants, the magnitude of which also did not appear to be influenced by the predictor.

**Table 4:** Results of the logistic regression (R^2^, bias and JND) for each participant (P).

P	Predictor	R^2^	Bias (deg)	JND (deg)
1	*ΔObj-PreVel*	0.80	1.69	−1.64
2	*ΔObj-PreVel*	0.91	1.45	−0.96
3	*ΔObj-PreVel*	0.58	−3.14	−2.87
4	*ΔObj-Eye*	0.95	−0.93	−0.88
5	*ΔObj-Eye*	0.70	0.64	−2.44
6	*ΔObj-PreVel*	0.33	−2.06	−7.07
7	*ΔObj-PreVel*	0.92	0.31	−1.04
8	*ΔObj-PreVel*	0.3	−5.66	−6.59
9	*ΔObj-PreVel*	0.89	0.12	−1.53
10	*ΔObj-PreVel*	0.67	−1.71	−2.24

Only data from the predictor with the highest R^2^ is shown. *ΔObj-PreVel* is the difference between object reappearance position and an extrapolation of the occluded object position based on pre-occlusion velocity. *ΔObj-Eye* is the difference between object reappearance position and eye position at reappearance.

## Discussion

The current experiment examined for the first time the accuracy of spatial estimation and eye movements in a linear prediction motion task (i.e., interruption paradigm) with accelerating objects. We used such a task because it is emphasizes extrapolation during occlusion and thereby could have a different informational basis than temporal prediction motion, where participants do not maintain ocular pursuit close to the occluded object and do not show sensitivity to the effects of acceleration in their temporal estimation [5]. We found that for spatial estimation there was a general bias, with participants being less likely to report the object to have reappeared behind than ahead of the expected position. Taking constant velocity trials as the baseline, the bias resulted in objects reappearing with a one degree negative position step being correctly estimated at approximately chance level. The implication is that participant’s extrapolation lagged behind the occluded object motion, thus leading to a tendency to underestimate the occluded distance [26]. In addition, for spatial estimation we found that participants were more likely to report negatively accelerating objects as reappearing behind the expected position than positively accelerating objects of equal magnitude. Logistic curve fitting on spatial estimation data plotted against the reappearance error that would have been evident had participants extrapolated the occluded trajectory based on pre-occlusion velocity indicated similar PSE for each level of object acceleration (Figure 5). This finding would not be expected if spatial estimation involved a comparison between object reappearance position and a veridical extrapolation of the occluded object motion. Consistent with recent work on temporal prediction motion, the spatial estimation data reported here indicate that the effects of acceleration are not taken into account by the perceptual system when extrapolating occluded object motion [5], [9], [27].

Although trials were received in random order, the duration of the initial visible part of the trajectory (i.e., 800 ms) was sufficient for participants to achieve good correspondence between eye and object motion at occlusion. Participants’ ocular response was reflective of veridical object motion characteristics, hence indicating that they were sensitive to the effects of acceleration (see below for a discussion of the process). Thereafter, and different to the pattern of eye movements exhibited in the temporal prediction motion task, where participants make a large amplitude saccade shortly after occlusion that moves the eyes to the cue representing the arrival location [2], [5], we found here that participants continued to follow the occluded object with a combination of smooth pursuit and small saccadic eye movements all the way up until object reappearance. This was expected given that participants did not know in advance where and when the object would reappear [in the interruption task], thus placing greater demand on extrapolation of the occluded trajectory [7]. Nonetheless, extrapolation of the occluded object was not ideal and as such eye position lagged behind the object at reappearance, except for those with negative acceleration (−8 and −4 deg/s^2^) where there was good match. Albeit with a constant undershoot, qualitatively it would seem that eye position was better matched to an extrapolation based on an average or final velocity estimate rather than use of veridical motion characteristics.

Undershoot of object velocity was also evident at the moment of reappearance but critically there was a significant linear increase in eye velocity as a function of object acceleration. The opposite pattern was observed at disappearance, where there was a negative slope as a function of increasing acceleration. As can be seen from group mean data in Figure 4B, although not as steep as expected for ideal extrapolation of veridical motion, a positive linear trend in eye velocity at reappearance would not be predicted by a direct extrapolation based on pre-occlusion velocity or an average velocity estimate, for example from the final 100 ms [21] of the visible trajectory prior to occlusion. This pattern was also observed at the individual-participant level, with a significant positive slope in the regression between eye velocity and object velocity at reappearance exhibited by 7 of the 10 participants. Of the remaining participants, only 1 had a negative slope but this was not significant. It was also noted that the difference between pre-occlusion and reappearance velocity was smaller for positive compared to negative (i.e., matched pairs) object accelerations. This is indicative of a greater recovery when pursuing positively accelerating objects, and thus the use of changing velocity during the initial visible trajectory to extrapolate the occluded motion.

In combination, we interpret the eye movement data at occlusion and reappearance as indicating that the ocular response was reflective of the effects of object acceleration [15], [18]. However, due to limitations in retinal and extra-retinal input (see below for more discussion), participants were unable to maintain accurate pursuit of the occluded object. Sub-optimal scaling of pursuit eye movements to accelerating objects during occlusion can in part be explained by a lack of acceleration sensitive cells in motion processing areas of visual cortex (MT/MST), and the resulting high discrimination thresholds. For instance, while reconstruction of an acceleration signal can be achieved from population coding of the velocity signal [16], [23], [24], [28], this is subject to a certain amount of noise; for potential mechanisms see [15], [21]. A consequence of high discrimination thresholds for acceleration is particularly evident when attempting to pursue object motion properties presented in random order [18]. In these cases, there is less opportunity for long range predictive mechanisms (i.e., representation of change in velocity by object acceleration developed over repeated trials and implicit advance knowledge on the relationship between pre-occlusion and post-occlusion velocity) to influence the ocular response [14]. Instead, participants rely on short-term prediction (i.e., within-trial) during the initial visible part of object motion. Somewhat akin to temporal integration of the velocity signal within a short temporal window [21], [23], we have proposed a model of ocular pursuit that includes a mechanism for sampling and storing velocity information [18], [29], [30]. The outcome of this process will be influenced by the sampled input (i.e., the changing velocity signal), but still participants could gain access to an implicit acceleration signal, which then exerts a weak but significant influence on the ocular response during occlusion. An alternative interpretation is that participants learned over repeated randomly-ordered trials that initial velocity or an average velocity estimate (during the final 100 ms) was negatively correlated with acceleration, and thus volitionally scaled their ocular response during occlusion in accord with this rule. While we tried to minimize the use of a simple heuristic (i.e., long range prediction) by randomizing trial order, we cannot discount this possibility. In fact, the use of an indirect strategy based on recognizing changing velocity of the accelerating objects (i.e., decreasing, constant or increasing) could also have been at work.

If one accepts that the effects of acceleration on object position and velocity were reflected in eye movements, and in particular leading up to occlusion, it remains to be considered why spatial estimation was consistent with an extrapolation based on pre-occlusion object velocity. Indeed, given the reported lower threshold of perception compared to ocular pursuit for sensitivity to acceleration [15], one might expect the opposite effect. In answering this question, it should be borne in mind that although early cortical processing (MT/MST) of visual motion stimuli such as velocity and acceleration [16] are common to perception and oculomotor control [31], there is evidence (neurophysiological and behavioral) for a divergence in processing downstream that can lead to different discrimination thresholds [15], or independent responses depending on the task at hand [32]. Therefore, although we found that the effects of object acceleration were apparent in the ocular response (disappearance and reappearance), and participants self-reported in unstructured post-test interviews that they were aware the objects did not all move with constant velocity, it does not necessarily follow that this should be reflected in the spatial estimation data. It could be the case that while an implicit acceleration signal was represented in the drive to ocular pursuit [30], [33], these properties were either not conveyed to the perceptual system [34], or were overridden by other sources of information (i.e., retinal and extra-retinal) available to participants after the object reappeared. It is possible that this was influenced by the limited representation of acceleration during randomly-order trials. That said, we believe that the continuation of eye movements during occlusion was not simply a mechanical carry-over effect from pursuit during the initial visible part of the trajectory. Indeed, findings from a similar prediction motion task indicated that participants exhibited more spatial estimation errors in a condition that demanded fixation compared to pursuit [2]. Based on the weight of recent evidence, we suggest that participants continued to move their eyes during occlusion in order to facilitate a visual discrimination between object reappearance position and the most recent and salient information for the goal of the task at hand; for a similar account see [35]. Not attempting to maintain pursuit would seem a somewhat unnatural response that could also result in image blur at reappearance. Here, logistic regression on individual participant data indicated that spatial estimation was predicted by an extrapolation based on pre-occlusion velocity. It will be interesting in future work to consider whether this holds across a wider range of velocities and accelerations, as well as duration of initial visible trajectory, because these factors are likely to influence perceptual and oculomotor sensitivity [16], [20].

### Conclusions

Our results showed a general bias in estimating the reappearance position of an occluded object in a prediction motion task. Moreover, for the majority of participants, spatial estimation was best predicted by an extrapolation based on object velocity at the moment of occlusion. Our finding of limited scaling of eye movements to accelerating objects is consistent with recent models of oculomotor control that sample and store the changing velocity signal. Together, these data add to the developing opinion that eye movements during occlusion contribute but do not uniquely specify information for accurate estimation (spatial or temporal) in prediction motion.
